# Supplementary material for: Artificial initiation codons and engineered initiator tRNAs enable N-terminal noncanonical amino acid incorporation in intact cell-free translation systems
Source: bioRxiv. 2026 May 25:2026.05.24.725928. Preprint. [Version 1] doi: 10.64898/2026.05.24.725928 (PMC13232202; doi:10.64898/2026.05.24.725928)
Supplement: Supplement 2 [file NIHPP2026.05.24.725928v1-supplement-2.pdf]

bioRxiv preprint doi: https://doi.org/10.64898/2026.05.24.725928; this version posted May 25, 2026. The copyright holder for this preprint (which was not certified by peer review) is the author/funder, who has granted bioRxiv a license to display the preprint in perpetuity. It is made available under a CC-BY 4.0 International license.

Supplementary information

Artificial initiation codons and engineered initiator tRNAs enable N-terminal noncanonical amino acid incorporation in intact cell-free translation systems

Haruyuki Furukawa[1], Yasunori Okamoto[2,3,4], and Naohiro Terasaka[1]*

1. Earth-Life Science Institute, Institute of Future Science, Institute of Science Tokyo, 2-12-1 Ookayama, Meguro-ku, Tokyo 152-8550, Japan
2. Research Center of Integrative Molecular Systems, Institute for Molecular Science, National Institutes of Natural Sciences, 5-1 Higashiyama, Myodaiji-cho, Okazaki, Aichi 444-8787, Japan
3. The Exploratory Research Center on Life and Living Systems (ExCELLS), National Institutes of Natural Sciences, 5-1 Higashiyama, Myodaiji-cho, Okazaki, Aichi 444-8787, Japan
4. SOKENDAI (The Graduate University for Advanced Studies), 5-1 Higashiyama, Myodaiji-cho, Okazaki, Aichi 444-8787, Japan

*Corresponding author,Contact: nterasaka@elsi.jp

bioRxiv preprint doi: https://doi.org/10.64898/2026.05.24.725928; this version posted May 25, 2026. The copyright holder for this preprint (which was not certified by peer review) is the author/funder, who has granted bioRxiv a license to display the preprint in perpetuity. It is made available under a CC-BY 4.0 International license.

Supplementary Methods 3
Supplementary Figures 6

bioRxiv preprint doi: https://doi.org/10.64898/2026.05.24.725928; this version posted May 25, 2026. The copyright holder for this preprint (which was not certified by peer review) is the author/funder, who has granted bioRxiv a license to display the preprint in perpetuity. It is made available under a CC-BY 4.0 International license.

Supplementary Methods

Preparation of linear DNA templates for IVTT and *in vitro* transcription

Linear DNA templates for *in vitro* translation and *in vitro* transcription were generated by PCR using plasmids as templates and appropriate primers. Amplified DNA fragments were purified using the FastGene Gel/PCR Extraction Kit (FG-91302, NIPPON Genetics) and quantified with the Qubit dsDNA Quantification Assay Kit (Q32854, Thermo Fisher Scientific). All sequences used in this study are provided in Supplementary Tables S1–S7. Specifically, DNA oligonucleotide sequences are listed in Supplementary Table S1, amino acid sequences of BindCraft-designed binders targeting $Brd4^{BD2}$ in Supplementary Table S2, amino acid sequences of individual proteins in Supplementary Table S3, nucleotide sequences of DNA templates for cell-free translation in Supplementary Table S4, DNA sequences of synthetic BD2 binder gene fragments used for fusion with mNG constructs in Supplementary Table S5, nucleotide sequences of DNA templates for in vitro transcription in Supplementary Table S6, and nucleotide sequences of $tRNA^{IniTx}$ in Supplementary Table S7.

T7pro-XXX-mNG (all 64 start codons): The mNG fragment was amplified from a plasmid using primers Oligo01 and Olig02, followed by agarose gel electrophoresis and gel extraction purification. The purified DNA fragment was then used as a template for a second PCR to introduce start codon variants and append a T7 promoter sequence. This reaction employed three primers (Oligo02, Oligo03, and Oligo04–67), where Oligo04–67 contained all 64 possible start codon sequences. PCR was performed using KOD One polymerase in a 20 μL reaction containing 10 ng template DNA, 0.3 μM Olig02, 0.3 μM Olig03, and 3 nM Oligo04–67. The cycling conditions were as follows: 98 °C for 10 s; 5 cycles of 98 °C for 10 s and 74 °C for 10 s; 5 cycles of 98°C for 10 s and 72 °C for 10 s; 5 cycles of 98 °C for 10 s and 70 °C for 10 s; followed by 20 cycles of 98 °C for 10 s and 68 °C for 10 s.

T7pro-CAC-mNG $Brd4^{BD2}$-targeting binder: The T7pro-CAC-mNG fragment was amplified from DNA template T7pro-CAC-mNG using Oligo68 and Oligo69 and purified by agarose gel electrophoresis followed by gel extraction. The BD2 binder fragment was synthesized as a gene fragment containing a 5′ overlap sequence for fusion to the mNG fragment and a 3′ sequence for downstream PCR amplification (see Supplementary Table

bioRxiv preprint doi: https://doi.org/10.64898/2026.05.24.725928; this version posted May 25, 2026. The copyright holder for this preprint (which was not certified by peer review) is the author/funder, who has granted bioRxiv a license to display the preprint in perpetuity. It is made available under a CC-BY 4.0 International license.

S2 for sequences). The T7pro-CAC-mNG fragment and the BD2 binder fragment were fused by overlap extension and amplified by PCR using Oligo01 and Oligo68.

T7pro-mScarlet-Brd4^{BD2}: The T7pro-mScarlet fragment was amplified from a plasmid using Oligo68 and Oligo70 and purified by agarose gel electrophoresis followed by gel extraction. The Brd4^{BD2} fragment was amplified from a linear DNA template (T7proN-SP6 RNAP-v4-BD2) described in the previous study[1] using Oligo01 and Oligo71 and purified by gel extraction. The T7pro-mScarlet fragment and the Brd4^{BD2} fragment were fused by overlap extension and subsequently amplified by PCR using Oligo01 and Oligo68.

T7pro-tRNA^{IniTx} variants: Five tRNA templates, tRNA$^{IniTx02}_{GGG}$, tRNA$^{IniTx02}_{GGA}$, tRNA$^{IniTx02}_{GUG}$, tRNA$^{IniTx03}_{GUG}$, and tRNA$^{IniTx04}_{GUG}$ were prepared by primer extension followed by PCR amplification. First, each template was assembled by primer extension using the following oligonucleotide pairs: Oligo72/Oligo73 for tRNA$^{IniTx02}_{GGG}$, Oligo72/Oligo74 for tRNA$^{IniTx02}_{GGA}$, Oligo72/Oligo75 for tRNA$^{IniTx02}_{GUG}$, Oligo76/Oligo77 for tRNA$^{IniTx03}_{GUG}$, and Oligo72/Oligo78 for tRNA$^{IniTX04}_{GUG}$. Primer extension was performed with each primer at a final concentration of 1 μM. The cycling conditions were as follows: 98 °C for 10 s; 5 cycles of 55 °C for 10 s and 68 °C for 10 s. The resulting products were then used directly as templates for PCR amplification in a 40-fold scaled reaction containing 1× KOD One Master Mix and 1 μM each of Oligo79 and Oligo80. Oligo80 contained 2′-O-methyl modifications at its two 5′-terminal nucleotides to suppress non-templated nucleotide addition by T7 RNA polymerase[2]. The cycling conditions were as follows: 98 °C for 10 s; followed by 25 cycles of 55 °C for 10 s and 68 °C for 10 s.

T7pro-T-boxzyme: Two types of T-boxzymes with distinct sequences were prepared to enable aminoacylation by recognizing tRNA anticodons GTG and GGA. These were Tx2.1CAC, which recognizes the GTG anticodon, and Tx2.1UCC, which recognizes the GGA anticodon. DNA templates for transcription of T-boxzymes were generated using Oligo81/Oligo82 for Tx2.1CAC and Oligo83/Oligo84 for Tx2.1UCC, followed by PCR amplification using Oligo85/Oligo86. Primer extension was performed with each primer at a final concentration of 1 μM. The cycling conditions were as follows: 98 °C for 10 s; 5 cycles of 55 °C for 10 s and 68 °C for 10 s. The resulting products were then used

bioRxiv preprint doi: https://doi.org/10.64898/2026.05.24.725928; this version posted May 25, 2026. The copyright holder for this preprint (which was not certified by peer review) is the author/funder, who has granted bioRxiv a license to display the preprint in perpetuity. It is made available under a CC-BY 4.0 International license.

directly as templates for PCR amplification in a 20-fold scaled reaction containing 1× KOD One Master Mix and 1 μM each of Oligo85 and Oligo86. The cycling conditions were as follows: 98 °C for 10 s; followed by 15 cycles of 55 °C for 10 s and 68 °C for 10 s.

bioRxiv preprint doi: https://doi.org/10.64898/2026.05.24.725928; this version posted May 25, 2026. The copyright holder for this preprint (which was not certified by peer review) is the author/funder, who has granted bioRxiv a license to display the preprint in perpetuity. It is made available under a CC-BY 4.0 International license.

Supplementary Figures

tRNA	$tRNA^{IniTx02}_{GGG}$	$tRNA^{IniTx02}_{GGA}$	$tRNA^{IniTx02}_{GUG}$	$tRNA^{IniTx03}_{GUG}$	$tRNA^{IniTx04}_{GUG}$
Secondary structure	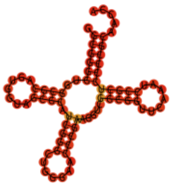	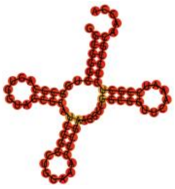	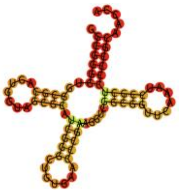	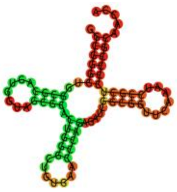	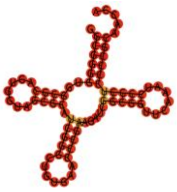
ΔG (kcal/mol)	-26.75	-32.15	-26.84	-27.26	-26.73
MFE structure frequency (%)	66.92	66.76	57.58	40.25	69.13

Figure S1. Predicted secondary structures and thermodynamic parameters of tRNA^{IniTx} variants. Secondary structures of the five designed tRNA^{IniTx} variants were predicted using RNAfold. Corresponding minimum free energy (MFE) values and predicted thermodynamic parameters are summarized alongside each structure.

bioRxiv preprint doi: https://doi.org/10.64898/2026.05.24.725928; this version posted May 25, 2026. The copyright holder for this preprint (which was not certified by peer review) is the author/funder, who has granted bioRxiv a license to display the preprint in perpetuity. It is made available under a CC-BY 4.0 International license.

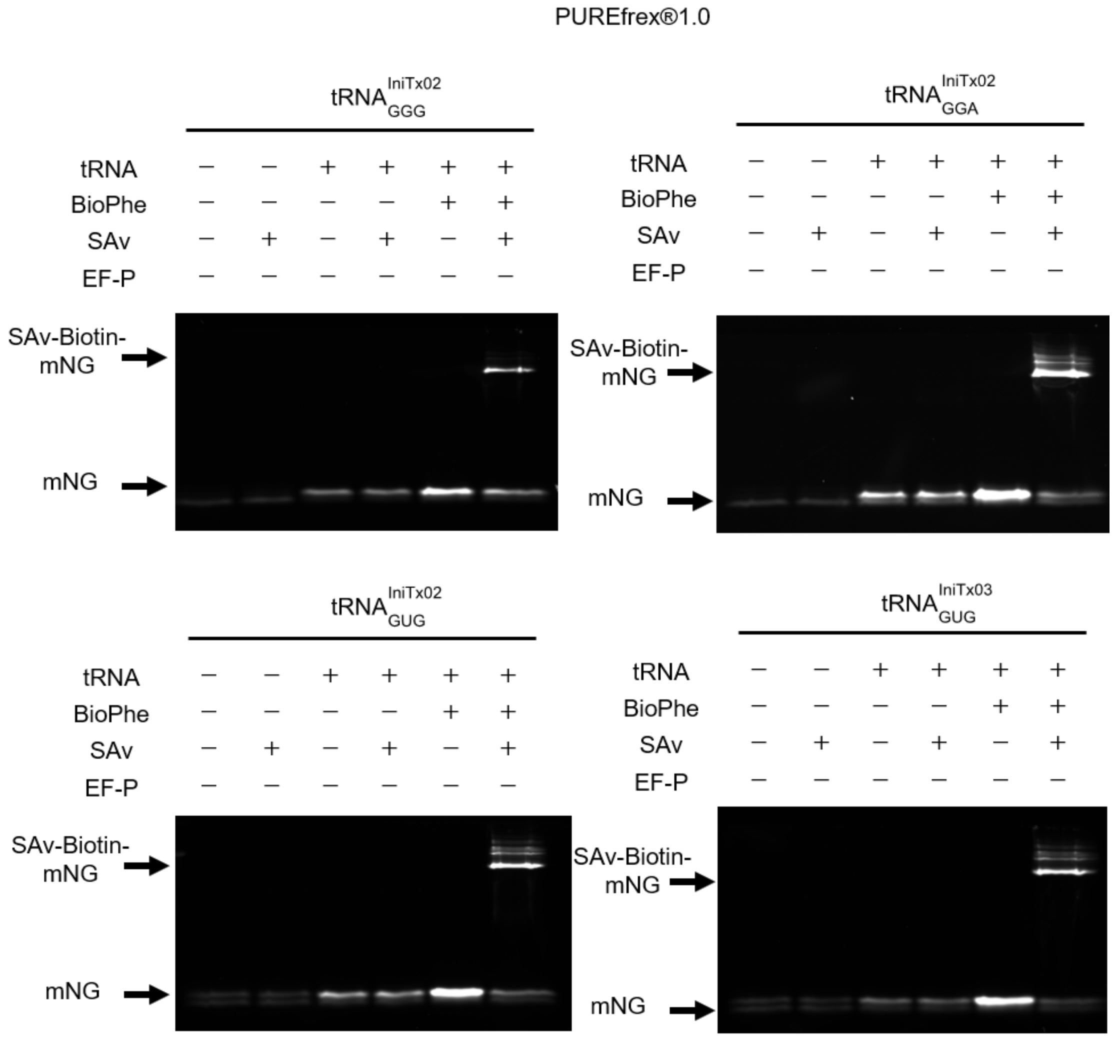

Figure S2. Detection of BioPhe incorporation into mNG in PUREfrex 1.0 without EF-P by SAv-dependent gel-shift assay. The gel was visualized based on the fluorescence of mNG in non-boiled SDS–PAGE.

bioRxiv preprint doi: https://doi.org/10.64898/2026.05.24.725928; this version posted May 25, 2026. The copyright holder for this preprint (which was not certified by peer review) is the author/funder, who has granted bioRxiv a license to display the preprint in perpetuity. It is made available under a CC-BY 4.0 International license.

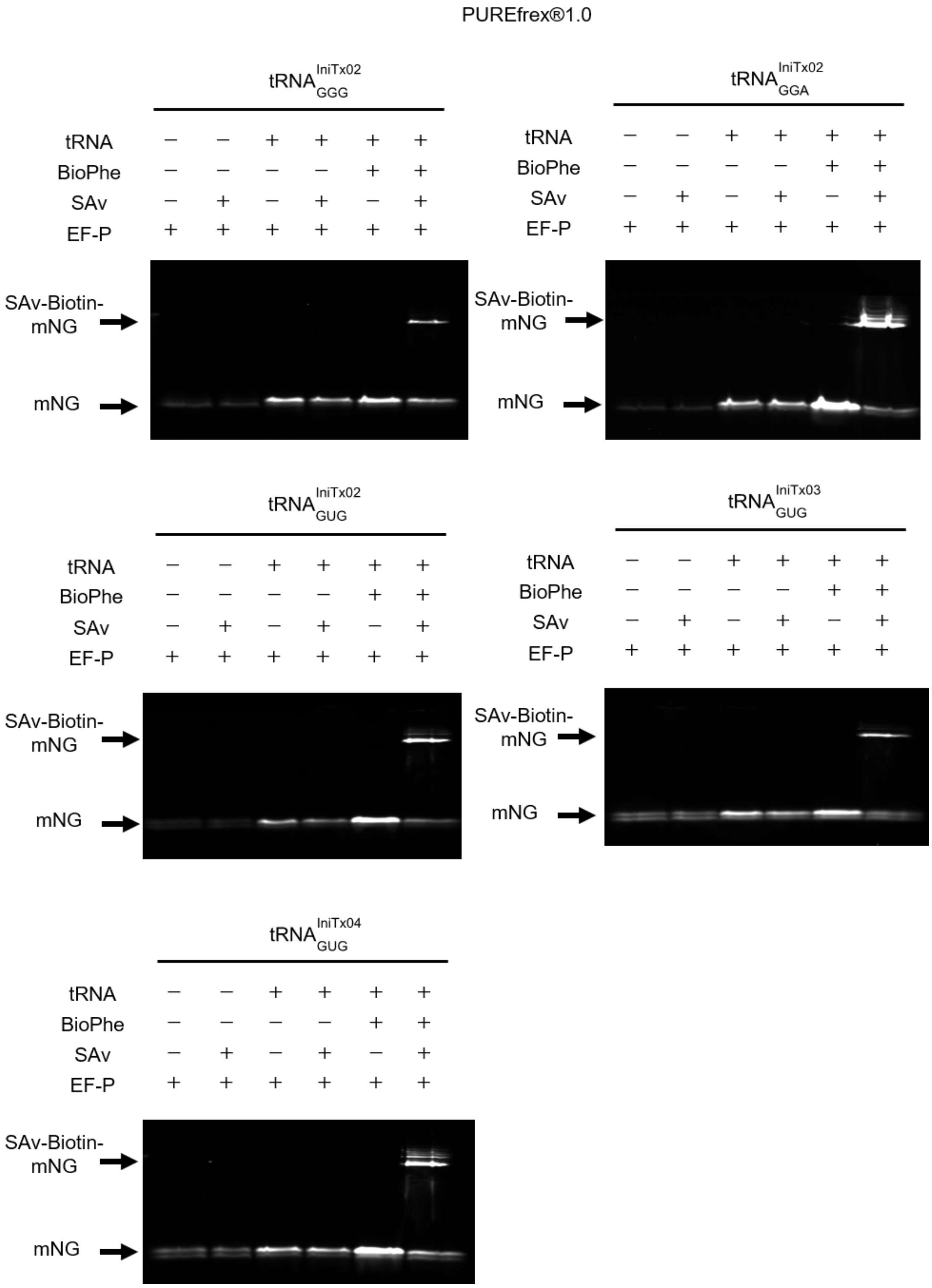

Figure S3. Detection of BioPhe incorporation into mNG in PUREfrex 1.0 with EF-P by SAv-dependent gel-shift assay. The gel was visualized based on the fluorescence of mNG in non-boiled SDS–PAGE.

bioRxiv preprint doi: https://doi.org/10.64898/2026.05.24.725928; this version posted May 25, 2026. The copyright holder for this preprint (which was not certified by peer review) is the author/funder, who has granted bioRxiv a license to display the preprint in perpetuity. It is made available under a CC-BY 4.0 International license.

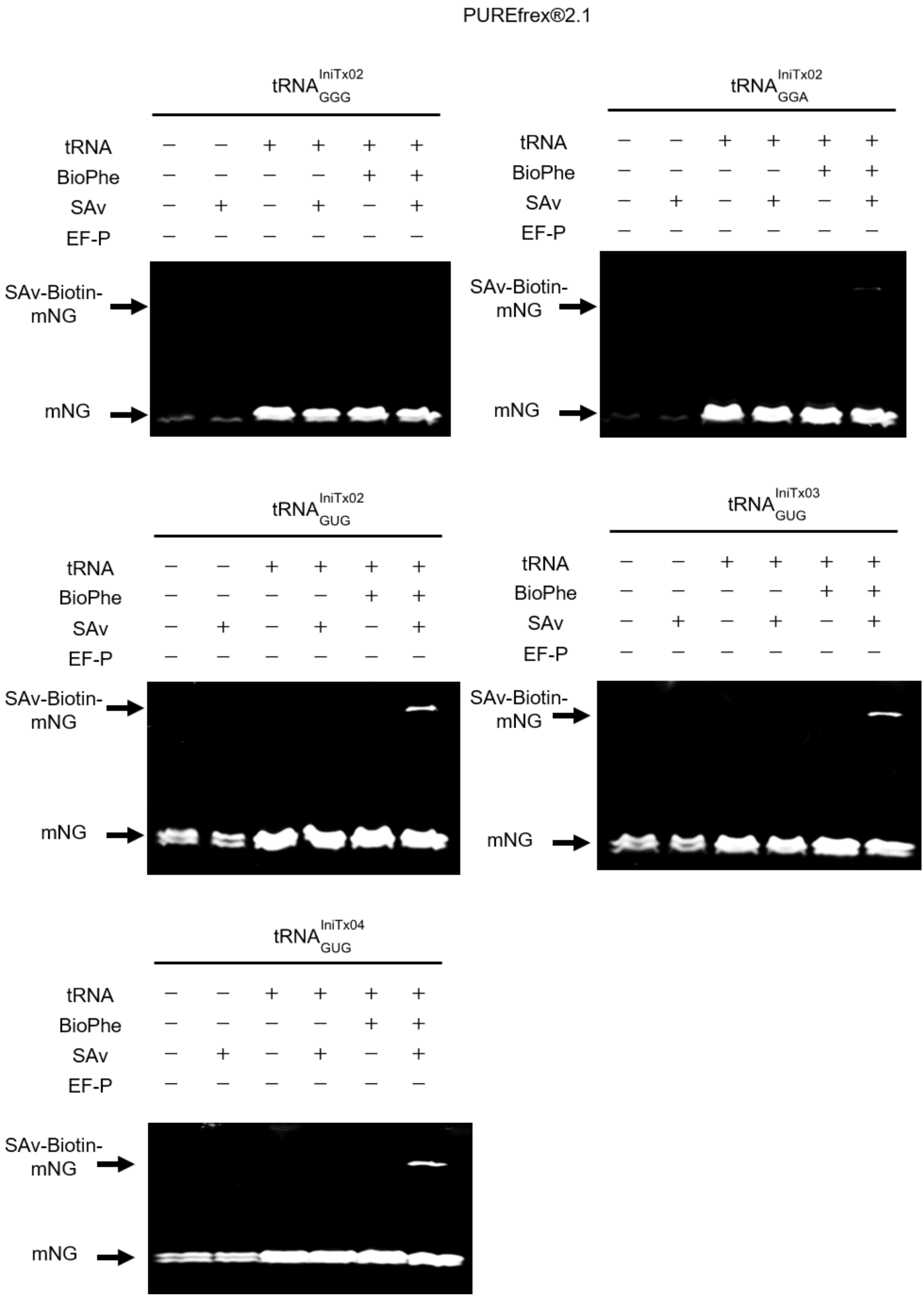

Figure S4. Detection of BioPhe incorporation into mNG in PUREfrex 2.1 without EF-P by SAv-dependent gel-shift assay. The gel was visualized based on the fluorescence of mNG in non-boiled SDS–PAGE.

bioRxiv preprint doi: https://doi.org/10.64898/2026.05.24.725928; this version posted May 25, 2026. The copyright holder for this preprint (which was not certified by peer review) is the author/funder, who has granted bioRxiv a license to display the preprint in perpetuity. It is made available under a CC-BY 4.0 International license.

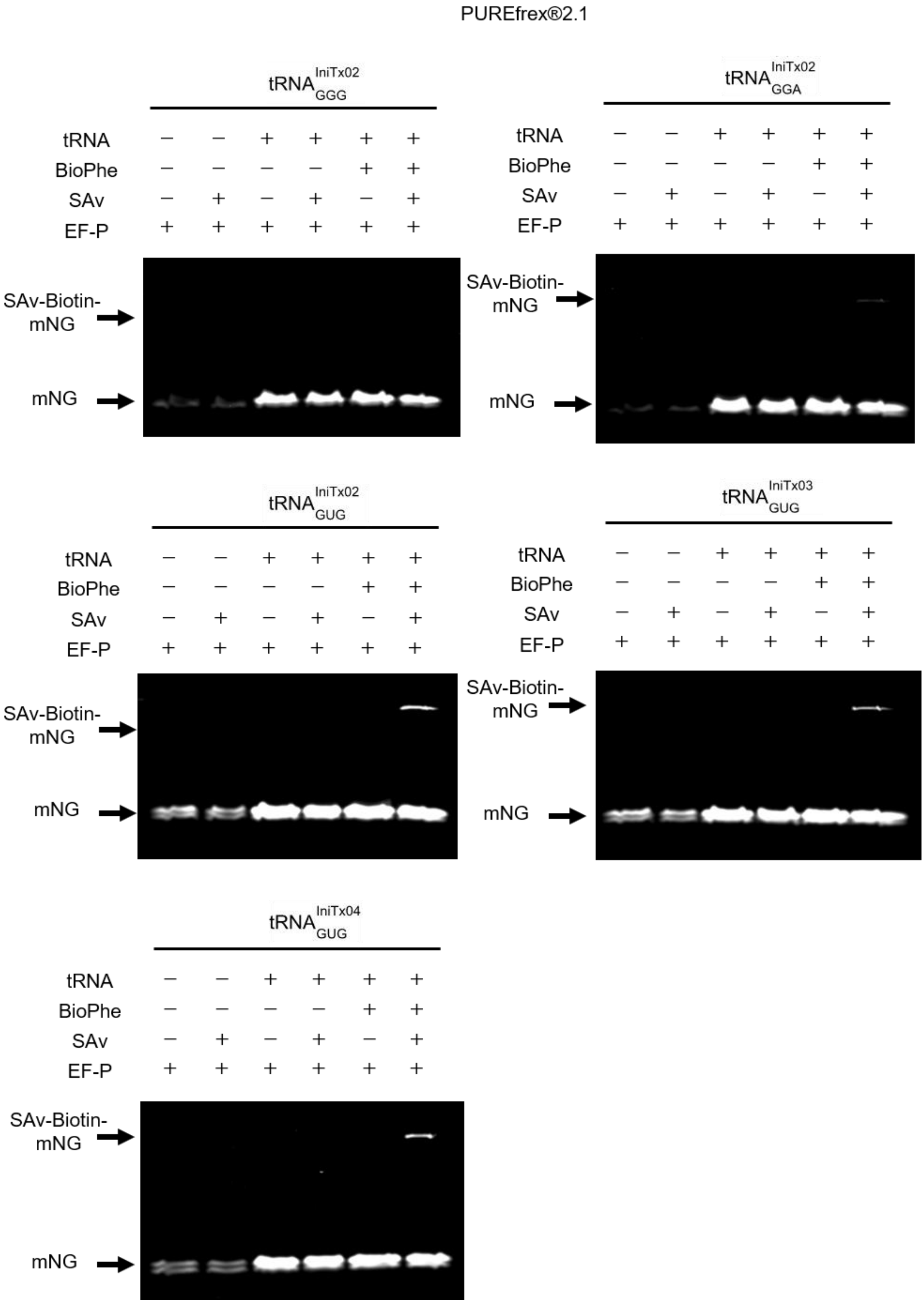

Figure S5. Detection of BioPhe incorporation into mNG in PUREfrex 2.1 with EF-P by SAv-dependent gel-shift assay. The gel was visualized based on the fluorescence of mNG in non-boiled SDS–PAGE.

bioRxiv preprint doi: https://doi.org/10.64898/2026.05.24.725928; this version posted May 25, 2026. The copyright holder for this preprint (which was not certified by peer review) is the author/funder, who has granted bioRxiv a license to display the preprint in perpetuity. It is made available under a CC-BY 4.0 International license.

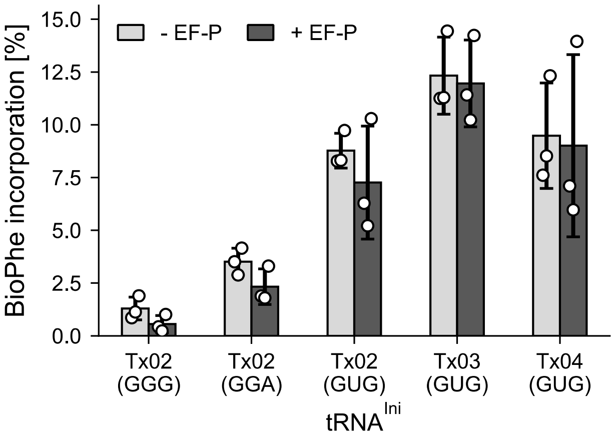

Figure S6. Comparison of BioPhe incorporation efficiencies among five tRNA^{IniTx} variants corresponding to artificial initiation codons in PUREfrex 2.1. The data represent the mean ± s.d. of three independent translation reactions (n = 3).

bioRxiv preprint doi: https://doi.org/10.64898/2026.05.24.725928; this version posted May 25, 2026. The copyright holder for this preprint (which was not certified by peer review) is the author/funder, who has granted bioRxiv a license to display the preprint in perpetuity. It is made available under a CC-BY 4.0 International license.

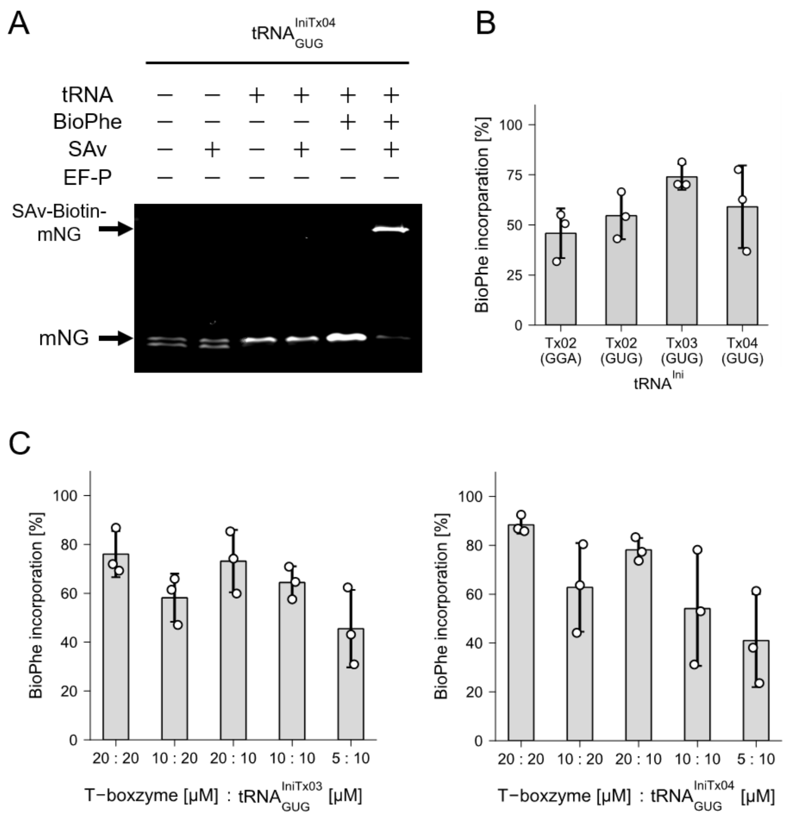

Figure S7. BioPhe incorporation by Tx2.1-mediated aminoacylation. (**A**) Detection of BioPhe incorporation using tRNA$^{IniTx04}_{GUG}$ into mNG by SAv gel-shift assay in non-boiled SDS–PAGE. (**B**) BioPhe incorporation efficiencies by four tRNA^{IniTx} variants cognate to selected artificial initiation codons. (**C**) Optimization of aminoacylation conditions by T-boxzyme for tRNA$^{IniTx03}_{GUG}$ (left panel) and tRNA$^{IniTx04}_{GUG}$ (right panel). Data in (B) and (C) represent the mean ± s.d. of three independent translation reactions (n = 3).

bioRxiv preprint doi: https://doi.org/10.64898/2026.05.24.725928; this version posted May 25, 2026. The copyright holder for this preprint (which was not certified by peer review) is the author/funder, who has granted bioRxiv a license to display the preprint in perpetuity. It is made available under a CC-BY 4.0 International license.

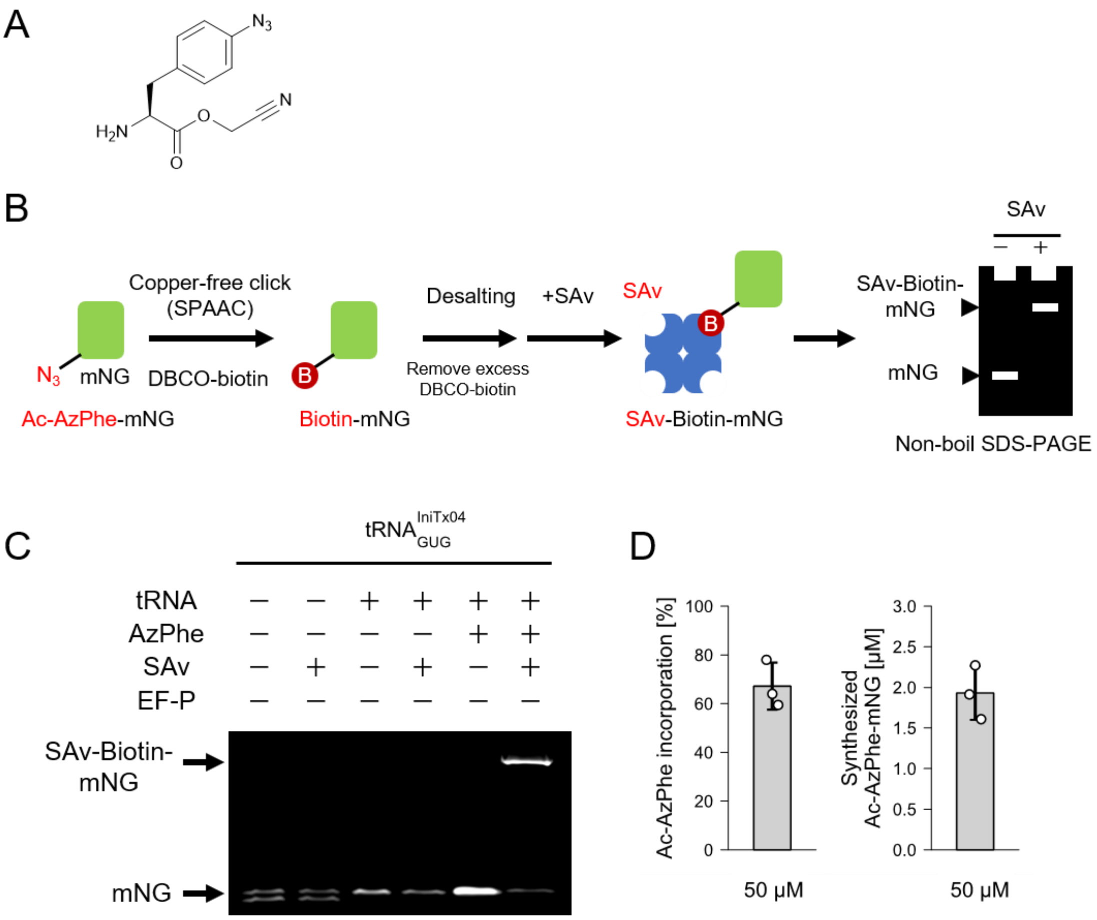

Figure S8. Evaluation of Ac-AzPhe incorporation using tRNA$^{\mathrm{IniTx04}}_{\mathrm{GUG}}$. (A) Chemical structure of AzPhe-CME. (**B**) Schematic representation of the workflow for detection of Ac-AzPhe-incorporated mNG. Ac-AzPhe-containing proteins were labeled with sulfo-DBCO-biotin via copper-free click chemistry, followed by desalting to remove unreacted DBCO-biotin. Biotinylated proteins were then incubated with streptavidin (SAv) and analyzed by non-boiled SDS–PAGE. (**C**) Detection of Ac-AzPhe incorporation using tRNA$^{\mathrm{IniTx04}}_{\mathrm{GUG}}$ into mNG by SAv gel-shift assay in non-boiled SDS–PAGE. (**D**) Quantification of Ac-AzPhe incorporation efficiency (left panel) and yield of Ac-AzPhe-mNG (right panel). Data represents the mean ± s.d. of three independent translation reactions (n = 3).

bioRxiv preprint doi: https://doi.org/10.64898/2026.05.24.725928; this version posted May 25, 2026. The copyright holder for this preprint (which was not certified by peer review) is the author/funder, who has granted bioRxiv a license to display the preprint in perpetuity. It is made available under a CC-BY 4.0 International license.

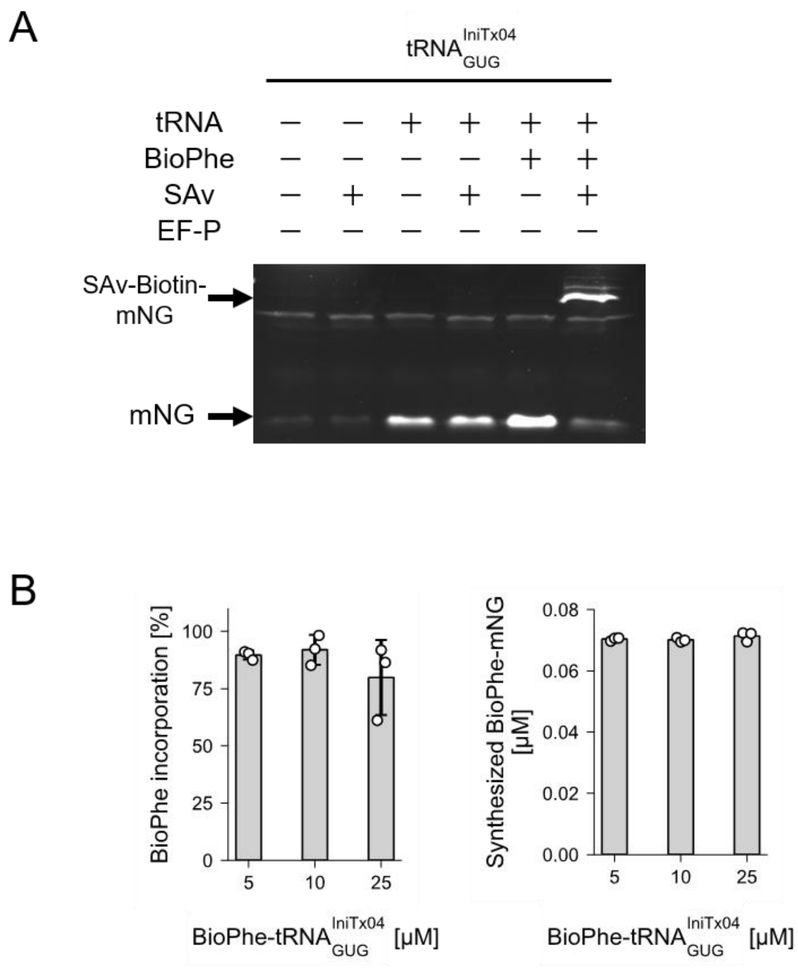

Figure S9. BioPhe incorporation in an *E. coli* extract–based cell-free translation system. (**A**) Detection of BioPhe incorporation using $tRNA^{IniTx04}_{GUG}$ into mNG by SAv gel-shift assay in non-boiled SDS–PAGE. (**B**) Effect of BioPhe-$tRNA^{IniTx04}_{GUG}$ concentration on incorporation efficiency (left panel) and absolute yield of BioPhe-incorporated mNG (right panel). Data represent the mean ± s.d. of three independent reactions (n = 3).

bioRxiv preprint doi: https://doi.org/10.64898/2026.05.24.725928; this version posted May 25, 2026. The copyright holder for this preprint (which was not certified by peer review) is the author/funder, who has granted bioRxiv a license to display the preprint in perpetuity. It is made available under a CC-BY 4.0 International license.

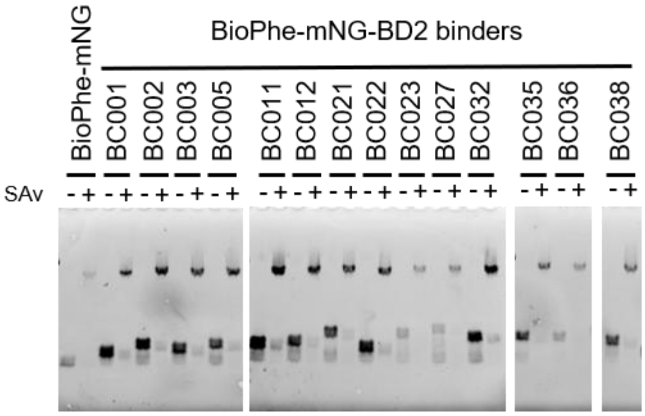

Figure S10. Expression analysis of BioPhe-mNG binders. Designed BioPhe-mNG-Brd4^{BD2} binders were expressed using PUREfrex 1.0 supplemented with BioPhe-tRNA$^{IniTx04}_{GUG}$. BioPhe incorporation into mNG was visualized by SAv gel-shift assay in non-boiled SDS–PAGE.

bioRxiv preprint doi: https://doi.org/10.64898/2026.05.24.725928; this version posted May 25, 2026. The copyright holder for this preprint (which was not certified by peer review) is the author/funder, who has granted bioRxiv a license to display the preprint in perpetuity. It is made available under a CC-BY 4.0 International license.

Reference

(1) Terasaka, N.; Furubayashi, T.; Tajima, K.; Noji, H. Efficient Cell-Free Evolution of RNA Polymerases by Droplet Microfluidics. *bioRxiv* February 23, **2026**, p 2026.02.23.707346. https://doi.org/10.64898/2026.02.23.707346.

(2) Kao, C.; Zheng, M.; Rüdisser, S. A Simple and Efficient Method to Reduce Nontemplated Nucleotide Addition at the 3′ Terminus of RNAs Transcribed by T7 RNA Polymerase. *RNA* **1999**, 5 (9), 1268–1272. https://doi.org/10.1017/S1355838299991033.
